# Comparison of Abbott ID Now and Abbott m2000 Methods for the Detection of SARS-CoV-2 from Nasopharyngeal and Nasal Swabs from Symptomatic Patients

**DOI:** 10.1128/JCM.00798-20

**Published:** 2020-07-23

**Authors:** Amanda Harrington, Brian Cox, Jennifer Snowdon, Jonathan Bakst, Erin Ley, Patricia Grajales, Jack Maggiore, Stephen Kahn

**Affiliations:** a Department of Pathology and Laboratory Medicine, Loyola University Medical Center, Maywood, Illinois, USA; b Department of Pathology and Laboratory Medicine, Cedars-Sinai Medical Center, Los Angeles, California, USA; Boston Children’s Hospital

**Keywords:** COVID-19, NAAT, diagnostic testing, point of care

## LETTER

The ID Now COVID-19 (IDNCOV) assay performed on the ID Now instrument (Abbott Diagnostics, Inc., Scarborough, ME) is a rapid diagnostic test that can be performed in a point-of-care setting equivalent to Clinical Laboratory Improvement Amendments (CLIA)-waived testing. The assay utilizes isothermal amplification and can reportedly deliver results in approximately 5 to 13 min. As this assay could provide significant improvements to workflow in our hospital system, we sought to compare the performance of this test with our current coronavirus disease 2019 (COVID-19) assay, the Abbott RealTime SARS-CoV-2 (severe acute respiratory syndrome coronavirus 2) (ACOV) assay performed on the Abbott m2000 system (Abbott Molecular Inc., Des Plaines, IL).

We compared the results from 524 paired foam nasal swabs (NS) tested on IDNCOV with nasopharyngeal swabs (NPS) placed in viral transport media tested on ACOV collected consecutively from symptomatic patients meeting current criteria for a diagnosis of COVID-19 (1). Five locations were included in the evaluation including three emergency departments (ED) and two immediate care centers (IMCC). IMCC A and ED 2 were experienced users of the IDNow platform. The other sites were new users of the platform and received training specifically for the IDNCOV. All ACOV testing was performed by one central clinical laboratory, and all NPS were heat inactivated for 30 min at 60°C prior to testing. NS were tested directly on the IDNCOV from IMCCs, and the tests were performed on-site. NS from the EDs were transported to the clinical microbiology laboratory in sterile transport containers (urine cups or conical tubes) and tested by laboratory personnel at each separate location. Statistical analysis was performed using SPSS v.26.

The overall positivity rate in this sample collection was 35%, ranging from 22% to 60% among the five sites. Overall agreement was 75% positive agreement (95% confidence interval [95% CI], 67.74%, 80.67%) and 99% negative agreement (95% CI, 97.64%, 99.89%) between IDNCOV and ACOV for all specimens tested. Agreement at individual sites varied (Table 1). Two subjects tested positive on IDNCOV that were initially negative on ACOV. In case one, a repeat sample was positive on ACOV (repeat IDNCOV was not performed), and the case was resolved as a true positive result. For case two, all repeat testing (both IDNCOV and ACOV) was negative and was resolved as a likely false-positive result. This sample was collected during the first day of testing and could have been operator error.

**TABLE 1:** Agreement between ACOV and IDNCOV

Site	Total no. of samples tested	No. of samples with the following result^*a*^:				% Positivity	Positive agreement (95% CI)	Negative agreement (95% CI)	Performance agreement (kappa) (95% CI)
		A+/IND+	A+/IND−	A−/IND+	A−/IND−				
IMCC A	208	33	13	1	161	22	71.74 (56.32, 83.54)	99.38 (96.09, 99.97)	0.783 (0.779, 0.788)
IMCC B	125	39	17	0	69	44	69.64 (55.74, 80.84)	100.0 (93.43, 100.0)	0.711 (0.706, 0.717)
ED 1	105	26	11	0	68	35	70.27 (52.83, 83.56)	100.0 (93.33, 100.0)	0.751 (0.744, 0.757)
ED 2	31	12	3	0	16	50	80.0 (51.37, 94.69)	100 (75.92, 100.0)	0.803 (0.792, 0.814)
ED 3	55	29	3	1	22	60	90.63 (73.83, 97.55)	95.65 (76.03, 99.77)	0.852 (0.844, 0.861)

Total	524	139	47	2	336	35	74.73 (67.74, 80.67)	99.41 (97.64, 99.89)	

a Positive (+) and negative (−) results by ACOV (A) and IDNCOV (IND) are shown.

**FIG 1:**
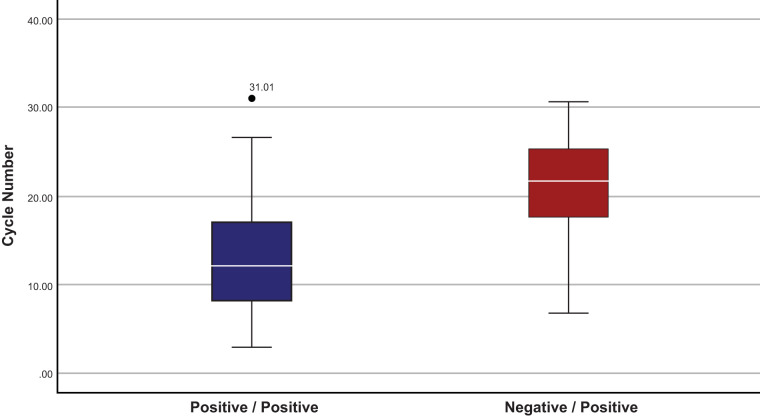
Boxplot of cycle numbers of concordant and discordant paired results. Distribution of cycle numbers from IDNCOV-positive/ACOV-positive samples (including a single data point [CN 31.01] outlier beyond the standard error) compared to INDCOV-negative/ACOV-positive samples.

Fleiss kappa analysis comparing the performance at each of the sites demonstrated that strength of agreement between the sites (Table 1) was rated as good to very good with comparable standard errors. We interpret this to mean that a site’s ability to run the test (or lack of experience) did not necessarily contribute to the variability in positivity that was found in this evaluation. Compared to the ACOV cycle numbers (CN) (which are similar but not directly comparable to cycle thresholds from other reverse transcription-PCR [RT-PCR] assays due to the unique ACOV assay design), a significant proportion, but not all, discordant samples exhibited at higher cycle numbers (Fig. 1). The mean CN for concordant positive samples was 12.71 (95% CI, 11.76, 13.67), ranging from 2.99 to 31.01, with a standard deviation of 5.5. The mean CN for discordant samples (ACOV positive [ACOV+]/IDNCOV negative [IDNCOV−]) was 21.07 (95% CI, 19.55, 22.60), ranging from 6.79 to 30.63, with a standard deviation of 5.1. These differences are statistically different (*P* = 6.75e−16). The stated limit of detection in the published instructions for use is 100 copies/ml for ACOV (2) and approximately 3,225 copies/ml when calculated based on the published genomes/reaction for IDNCOV (3). Based on the distribution of cycle numbers seen in Fig. 1 and performance agreement among the sites, negative results on IDNCOV are likely related to both a higher limit of detection on IDNCOV and preanalytical sampling error.

Overall, the ID Now COVID-19 assay demonstrated significantly different performance characteristics compared to the Abbott RealTime SARS-CoV-2 assay.
